# Gene expression and brain imaging association study reveals gene signatures in major depressive disorder

**DOI:** 10.1093/braincomms/fcae258

**Published:** 2024-08-13

**Authors:** Wei Liu, Jian-Po Su, Ling-Li Zeng, Hui Shen, De-Wen Hu

**Affiliations:** College of Intelligence Science and Technology, National University of Defense Technology, Changsha, Hunan 410073, P.R. China; College of Intelligence Science and Technology, National University of Defense Technology, Changsha, Hunan 410073, P.R. China; College of Intelligence Science and Technology, National University of Defense Technology, Changsha, Hunan 410073, P.R. China; College of Intelligence Science and Technology, National University of Defense Technology, Changsha, Hunan 410073, P.R. China; College of Intelligence Science and Technology, National University of Defense Technology, Changsha, Hunan 410073, P.R. China

**Keywords:** gene expression, brain imaging, major depression disorder, temporal–spatial expression specificity

## Abstract

Major depressive disorder is often characterized by changes in the structure and function of the brain, which are influenced by modifications in gene expression profiles. How the depression-related genes work together within the scope of time and space to cause pathological changes remains unclear. By integrating the brain-wide gene expression data and imaging data in major depressive disorder, we identified gene signatures of major depressive disorder and explored their temporal–spatial expression specificity, network properties, function annotations and sex differences systematically. Based on correlation analysis with permutation testing, we found 345 depression-related genes significantly correlated with functional and structural alteration of brain images in major depressive disorder and separated them by directional effects. The genes with negative effect for grey matter density and positive effect for functional indices are enriched in downregulated genes in the post-mortem brain samples of patients with depression and risk genes identified by genome-wide association studies than genes with positive effect for grey matter density and negative effect for functional indices and control genes, confirming their potential association with major depressive disorder. By introducing a parameter of dispersion measure on the gene expression data of developing human brains, we revealed higher spatial specificity and lower temporal specificity of depression-related genes than control genes. Meanwhile, we found depression-related genes tend to be more highly expressed in females than males, which may contribute to the difference in incidence rate between male and female patients. In general, we found the genes with negative effect have lower network degree, more specialized function, higher spatial specificity, lower temporal specificity and more sex differences than genes with positive effect, indicating they may play different roles in the occurrence and development of major depressive disorder. These findings can enhance the understanding of molecular mechanisms underlying major depressive disorder and help develop tailored diagnostic and treatment strategies for patients of depression of different sex.

## Teaser

Liu *et al*. reported an association analysis method of gene expression and brain imaging to identify depression-related genes and found their expression changes are related with the coordinated changes of grey matter density and functional activities in limited brain regions across multiple periods, with obvious sex differences.

## Graphical Abstract

**Graphical Abstract:**
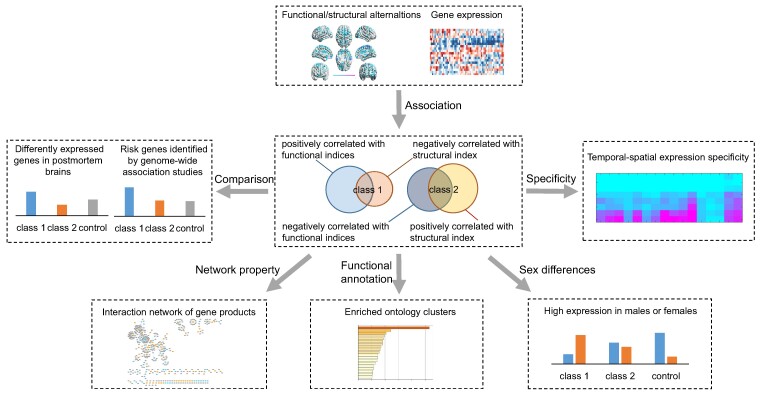

## Introduction

Major depressive disorder (MDD) is a prevalent psychiatric condition that affects over 350 million individuals globally, imposing a significant personal strain on those affected, as well as exerting a substantial economic burden on society.^1^ The aetiology of MDD is intricate, arising from the complex interplay between numerous environmental factors, genetic variations and alterations in gene expression patterns.^2-4^ Using different modalities of MRI, structural and functional alterations in the brain have been reported in patients with MDD,^5-7^ but there is still a lack of in-depth understanding of underlying molecular and cellular mechanisms.

Recent advancements in comprehensive brain-wide gene expression atlases, such as the Allen Human Brain Atlas (AHBA),^8^ have opened up the possibility of associating spatial variations in gene expression with macroscopic neuroimaging phenotypes.^9,10^ The methodological framework for integrating imaging and genomic data has been well established^11,12^ and utilized in the context of neuropsychiatric disorders.^13-16^ Some studies have employed the partial least square (PLS) analyses to discern a linear combination of genes that exhibit a similar cortical expression pattern to the map of volumetric changes observed in MDD in prior research.^17^ Other studies have conducted integrative omics analysis to identify transcriptome signatures.^18,19^ These researches suggested common and distinct genetic modulations of brain structural and functional impairments in MDD.^20,21^ However, most studies have not separated gene signatures of MDD by directional effects on the functional and structural changes.

Gene expression can exhibit extensive temporal–spatial variation across different tissues, developmental stages, physiological conditions and individuals.^22,23^ The temporal–spatial preference of the characteristic genes associated with MDD provides critical insights into the functions of genes and how they interact to perform specific physiological roles. Moreover, previous studies have reported differences between the genders in the structures and function connectivities in MDD supported by the neuroimaging analysis.^24-26^ A reasonable assumption is that there may exist sex differences in the temporal–spatial expression of genes related to MDD compared with the control genes. Therefore, examining temporal–spatial expression patterns of depression-related genes in the human brain is crucial for a thorough comprehension of the underlying mechanisms of MDD onset, genetic susceptibility to MDD and the difference in incidence rate between male and female patients.

**Figure 1:**
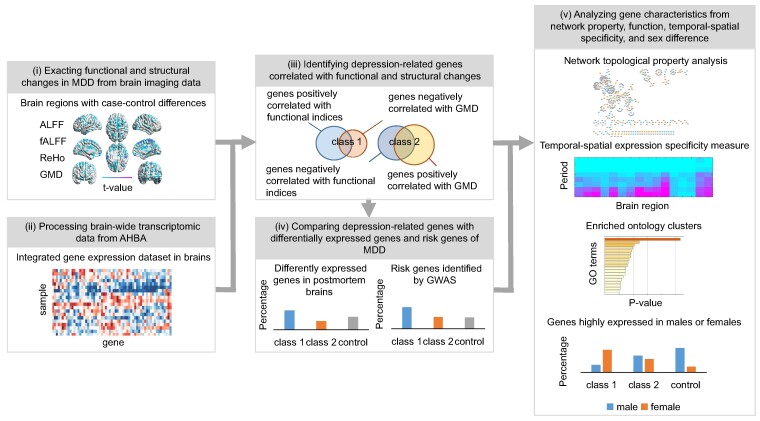
**Schematic representation of a general workflow for association analysis of gene expression and brain imaging.** The basic workflow involves: (i) extracting functional and structural changes in MDD from MRI data, to measure inter-group differences between patients with MDD and healthy controls; (ii) processing brain-wide transcriptomic data from AHBA to obtain a sample × gene expression data matrix; (iii) identifying depression-related genes correlated with functional or structural changes by combing neuroimage and gene expression data; (iv) comparing depression-related genes with differentially expressed genes in post-mortem brains of patients with MDD and depression risk genes identified by GWAS; (v) analysing characteristics of depression-related genes from multiple aspects, including network properties, temporal–spatial expression specificity, function annotations and sex differences. AHBA, Allen Human Brain Atlas; GWAS, genome-wide association studies; MDD, major depressive disorder.

The aim of our work is to devise a methodology that integrated the analysis of gene expression and brain imaging to identify gene signatures associated with MDD, distinguishing directional effects, and to systematically investigate their temporal–spatial expression specificity, network properties, function annotations and sex differences. To achieve this, we conducted an exhaustive correlation analysis of gene expression and brain imaging data to discern gene signatures and assessed their expression specificity across various brain regions in different periods. Initially, we identified inter-group alterations between patients with MDD and healthy controls using functional MRI (fMRI) and MRI data sets. We then procured sample-wise gene expression data across six post-mortem adult human brains available in AHBA. Following this, we employed correlation analysis to identify gene signatures whose transcriptional profiles are significantly correlated with the case–control changes in functional and structural indices. Then, we compared them with differently expressed genes in post-mortem brains of patients with MDD and depression risk genes identified by genome-wide association studies (GWASs). Finally, we analysed the network properties, temporal–spatial expression specificity, functional annotations and sex differences of depression-related genes, drawing upon the gene expression data of developing human brains from the Allen Brain Atlas. A schematic representation of the study’s protocol is presented in Fig. 1.

## Materials and methods

### Brain imaging data of MDD

The brain imaging data employed in this study were derived from the REST-meta-MDD project (http://rfmri.org/REST-meta-MDD).^27^ This initiative was launched to investigate the functional and structural changes in the brain induced by MDD, leveraging a substantial repository of brain image data. The consortium contributed 2428 previously gathered data sets (1300 patients with MDD and 1128 healthy controls) from 25 research groups across 17 hospitals. Some subjects were excluded due to bad imaging data and bad spatial normalization or other exclusion criterions. Finally, data of 848 patients with MDD and 794 normal controls were used for analysis. Comprehensive imaging and demographic details are accessible on the project’s website.

### Allen Human Brain Atlas

For gene expression data, publicly accessible information from six post-mortem human donors (*n* = 1 female), ranging in age from 24 to 57 years [M = 42.5, standard deviation (SD) = 13.38], were sourced from the AHBA, following the updated microarray normalization pipeline implemented in March 2013 (http://human.brain-map.org).^8^ Notably, the AHBA provides expansive coverage of nearly the entire brain, encompassing normalized expression data for 20 737 genes with unique Entrez IDs, detected by 58 692 probes from 3702 spatially distinct tissue samples. The establishment of spatial correspondence between gene expression and neuroimaging phenotypes was feasible in MNI space.

We amalgamated the gene expression data from all six brains employing the standard workflow for processing brain-wide transcriptomic data as delineated in an earlier publication.^11^ By aligning the samples to the cerebral cortex of human brains, we compiled an integrated data set comprising 15 745 genes expressed across 1280 samples. The following probe filtering criteria were applied during the process: (i) probe-to-gene annotations were updated utilizing the Re-Annotator package; (ii) probes where expression measures did not surpass the background in >50% samples were eliminated; (iii) a representative probe for each gene was chosen based on the peak intensity; (iv) the limma batch effect removal was applied to cross-subject aggregated data, succeeded by the scaled robust sigmoid normalization. Subsequent to normalization, the samples manifested no inter-individual disparities in gene expression. The gene expression levels were *z*-score normalized by subtracting the mean and dividing by the SD across all the samples.

### Ex vivo MDD patient differential expression

The estimates of differential expression from cortical expression data from patients with MDD were analysed.^28^ Post-mortem brain tissues, including pre-frontal cortex, hippocampus and striatum, were collected from control subjects and well-matched subjects with MDD (17 case samples and 19 control samples for each group). Gene expression values were normalized and then used for differential expression calculation by two-sided *t*-test, to obtain the degree that a gene is up- or downregulated for MDD. The differentially expressed gene list (*P* < 0.01) is available in Supplementary Table 1. Gene-wise patient differential expression was then correlated to the gene-wise spatial correlation to *in vivo* neuroimaging phenotypes.

### Risk genes identified in GWASs of depression

In a seminal study, Howard *et al*. undertook an extensive GWAS of depression (total *n* = 807 553), aggregating data from three preceding investigations on the subject.^3,29,30^ This cumulative analysis encompassed a total sample size of 807 553 individuals. The research identified 102 independent variants and 269 associated genes with the incidence of depression.^31^ We compared depression-related genes with these risk genes, and the overlap genes are available in Supplementary Table 2.

### Topological properties of networks

The degree of a node represents its most fundamental attribute, denoting the quantity of connections it maintains with other nodes within the network.^32^ Given the multitude of potential routes between any two nodes, the shortest path—defined by the least number of intermediary links between the selected nodes—holds significant importance. The betweenness centrality of a node is determined by the count of these shortest paths originating from all nodes to every other node, which traverse through the given node. A higher betweenness centrality indicates an increased capacity of the node to influence the dissemination of information throughout the network.^33^ It is postulated that cellular functions are predominantly executed in a highly modular fashion. The clustering coefficient serves as a metric to assess the propensity of a node to participate in clusters or groups, reflecting its tendency to form cohesive units within the network structure.^34^

### BrainSpan Atlas

As mentioned previously, we obtained the gene expression data pertaining to the developing human brains from the Allen Brain Atlas (http://www.brainspan.org).^35^ The study included only brains from donors who were clinically unremarkable and devoid of large-scale genomic abnormalities (*n* = 41; age, 8 post-conceptual weeks to 40 years; sex, 22 males and 19 females). We established an eight-period system that spans from embryonic development to adulthood (Supplementary Table 3). For our investigation, we utilized 547 transcriptome samples across 16 distinct brain regions, which included the cerebellar cortex, mediodorsal nucleus of the thalamus, striatum, amygdala, hippocampus and 11 neocortex areas (Supplementary Table 4). Comprehensive details regarding data pre-processing and normalization methodologies are accessible on the BrainSpan Atlas website (http://help.brain-map.org//display/devhumanbrain/Documentation).

### The measure for evaluating temporal–spatial specificity of genes

The specificity index (SPI) was employed to quantitatively assess the relative expression specificity of a gene within a given sample.^36^ Initially, each gene expression profile underwent a transformation into a vector *X*, as follows:


$$X=(x_{1},x_{2},\ldots ,x_{i},\ldots ,x_{n-1},x_{n})$$

where *n* is the total number of samples in the profile. Concurrently, a vector $X_{i}$ was formulated to depict the gene expression in an individual sample *i*:


$$X_{i}=(0,0,\ldots ,x_{i},\ldots ,0,0)$$

Subsequently, the SPI of a gene in a particular sample was ascertained by computing the cosine value of the intersecting angle *θ* between vector $X_{i}$ and *X* in a high-dimensional feature space, as follows:


$$\mathrm{SP}I_{i}=\cos\theta =\frac{X_{i}\times X}{\left|X_{i}|\times |X\right|}$$

where $\left|X_{i}\right|$ and |X| denote the length of vectors $X_{i}$ and *X*, respectively. An SPI value approaching 1.0 signifies that the gene expression in a specific sample (e.g. vector $X_{i}$) constitutes a significant proportion of its expression across all samples (vector *X*). The greater the SPI value, the more specific the gene expression within that sample.

Utilizing SPI, we introduced the dispersion index (DPI) as a metric to evaluate the specificity degree of a gene expression profile during a certain period or within a designated region. The gene expression profile (*X*) was transmuted into its corresponding SPI profile ($X_{\mathrm{SPI}}$), as follows:


$$X_{\mathrm{SPI}}=(\mathrm{SP}I_{1},\mathrm{SP}I_{2},\ldots ,\mathrm{SP}I_{i},\ldots ,\mathrm{SP}I_{n-1},\mathrm{SP}I_{n})$$

The DPI was then derived through the following equation:


$$\mathrm{DPI}=\sqrt{\frac{\sum_{i=1}^{n}\;{\left(\mathrm{SP}I_{i}-\bar{\mathrm{SPI}}\right)}^{2}}{n-1}}\times \sqrt{n}$$

where *n* signifies the sample number, and SPI signifies the mean of SPIs in a gene expression profile. In contrast to conventional SD analyses, DPI is unaffected by the gene expression level and the number of samples, as it scales to a region of 0–1.0, as mentioned previously. By this method, DPI facilitates the comparison of variability across profiles or data sets. The temporal–spatial specificity of genes can be quantitatively evaluated by considering the gene expression of samples in a period and a region as a profile, respectively. A value of DPI nearing 0 suggests a gene’s equal expression across samples. Conversely, a higher DPI value indicates a gene’s expression is more specific to a particular period or region. Thus, DPI serves as an effective indicator for the quantitative description and identification of genes that are overexpressed during a certain period or within a specific region. The temporal and spatial DPI values of the depression-related genes were computed and are presented in Supplementary Table 5.

### Sex difference in expression of depression-related genes

We identified differentially expressed genes with sex difference based on the gene expression data of developing human brains. We compared the expression levels of genes in the samples of the males and females with corresponding periods by two-sided *t*-test, and found 17 depression-related genes with sex difference in expression (*P* < 0.01), available in Supplementary Table 6. Then, we counted the percentage of genes highly expressed in males and females occupying the genes with sex difference, respectively.

### Statistical analysis

The inter-group difference of low frequency fluctuation (ALFF), fractional amplitude of low frequency fluctuation (fALFF), regional homogeneity (ReHo) and grey matter density (GMD) between patients with MDD and healthy controls were determined by two-sided *t*-tests. The statistical threshold of multiple comparison correction using the Benjamini–Hochberg method for all functional and structural indices is *P* < 0.001, corrected. We computed the Spearman correlation coefficient of spatial expression levels of genes and the case–control differences in functional indices and GMD between patients with MDD and healthy controls. To investigate whether the gene expression levels exhibited significant correlations with functional or structural indices than chance, we used 10 000 random permutations of the same sample size to derive permutation *P*-values and identified significantly correlated genes with *P* < 0.001. *P*-values for the spatial DPI values and temporal DPI values of depression-related genes compared with those of control genes were calculated using a two-sided *t*-test, with a statistical significance threshold set at 0.001. *P*-values for the expression levels of depression-related genes across various periods and regions were computed using a two-sided *t*-test, to investigate whether depression-related genes are significantly overexpressed relative to control genes from random chance, with a statistical threshold of 0.01. Accumulative hypergeometric *P*-values after multi-test adjustments and enrichment factors were computed and utilized for filtering (*P* < 0.01, a minimum count of 3 and an enrichment factor >1.5), to identify enriched functional annotation terms for a given gene set. Through a two-sided *t*-test in the expression data of the males and females through all periods, we identified genes with sex differences in expression, with *P* < 0.01. *P*-values for the expression levels of depression-related genes with sex differences in periods and regions were determined by conducting a two-sided *t*-test, to assess whether they are significantly different from those of control genes, with a statistical threshold of 0.01.

## Results

### Identification of functional and structural changes in MDD based on brain imaging analysis

The whole-brain resting-state functional indices and voxel-based morphometry analysis were used to identify the patterns of functional and structural alterations in MDD relative to healthy controls, utilizing brain imaging data of MDD (for details, see the Materials and methods section). For fMRI data, we extracted three functional indices, namely, ALFF, fALFF and ReHo^37^, to characterize brain functionality. For structural MRI data, we calculated GMD using normalized grey matter volumes extracted by Computational Anatomy Toolbox 12. The inter-group differences of ALFF, fALFF, ReHo and GMD between patients with MDD and healthy controls were assessed by a two-sided *t*-test.

**Figure 2:**
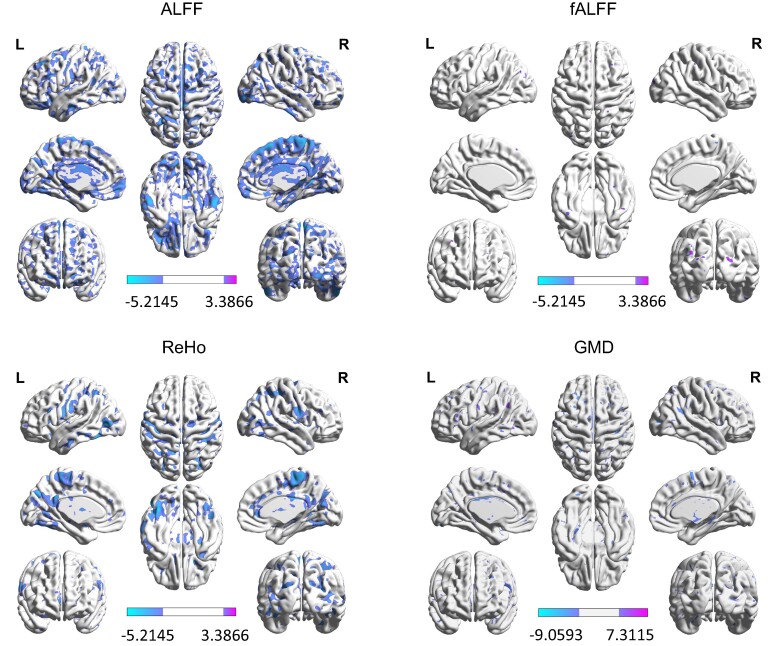
**Visualization of differential brain regions between patients with MDD and healthy controls in two-sided *t*-tests.** *T*-values for *t*-tests are indicated by colours, with *P* < 0.001, corrected. Positive values indicate higher indices in patients with MDD than controls and negative values indicate lower indices in patients with MDD than controls. Altogether 848 patients with MDD and 794 healthy controls are used in this study. ALFF, low frequency fluctuation; fALFF, fractional amplitude of low frequency fluctuation; GMD, grey matter density; ReHo, regional homogeneity.

We mapped the *t* statistics representing the case–control differences in functional indices and GMD at each cortical area (see Fig. 2). From the result of the statistical analysis, we found that the MDD group showed significantly altered GMD compared with the healthy control group in the temporal gyrus, especially in the temporal pole and fusiform gyrus. Furthermore, sparsely distributed between-group difference can also be found all around the cerebral cortex and cerebellum. Significant between-group difference is found all over the brain considering ALFF. While in fALFF, between-group difference is found in bilateral precuneus and paracentral gyrus, which is similar to the finding of ReHo. The detailed information on the regions with significant group differences in each of the functional indices and GMD is given in Supplementary Table 7.

### Identification of depression-related genes based on the association analysis of gene expression and brain imaging

By integrating the expression data of AHBA and functional and structural indices for neuroimaging, we computed the Spearman correlation coefficient between spatial expression levels of genes and the case–control differences in functional indices and GMD among MDD patients and healthy controls. To determine whether gene expression levels were significantly correlated with functional or structural indices beyond chance, we employed 10 000 random permutations of the same sample size to derive permutation *P*-values. As a result, we identified 345 depression-related genes, whose spatial expression is simultaneously correlated with case–control differences in functional indices and GMD, with permutation *P* < 0.001 for ALFF or fALFF or ReHo, and GMD.

**Figure 3:**
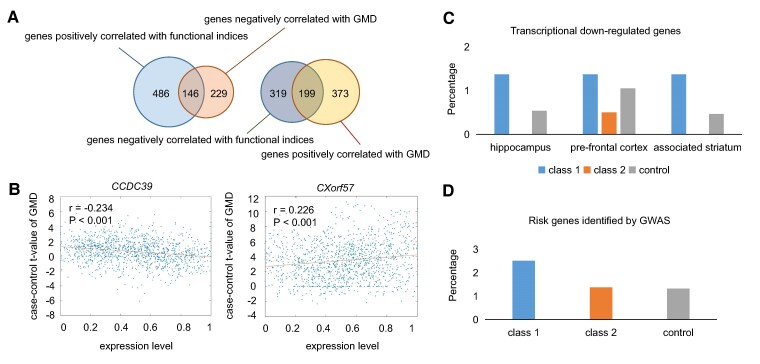
**The depression-related genes correlated with case–control differences in functional and structural indices between patients with MDD and healthy controls.** (**A**) The overlap of genes correlated with functional changes and those with structural changes in MDD. (**B**) The expression levels of *CCDC39* and *CXorf57* significantly correlated with GMD changes in MDD. For convenience, expression levels of *CCDC39* and *CXorf57* are normalized to between 0 and 1. (**C**) The percentage of downregulated genes in the samples of hippocampus, prefrontal cortex and associated striatum occupying the depression-related genes. The number of genes significantly downregulated in the samples of hippocampus, prefrontal cortex and associated striatum is 622, 227 and 368, respectively. (**D**) The percentage of depression risk genes occupying the genes in Class 1, Class 2 and controls. The number of depression risk genes in Class 1, Class 2 and controls is 5, 2 and 54, respectively. GMD, grey matter density; GWAS, genome-wide association studies; MDD, major depressive disorder.

Considering the gene signatures positively correlated and negatively correlated with functional and structural indices may play different functions in MDD, we separated them into two classes by directional effects and compared their expression characteristics with those of control genes. To separate depression-related genes by directional effects, we divided them into two categories: Class 1: genes positively correlated with functional indices and negatively correlated with GMD; Class 2: genes negatively correlated with functional indices and positively correlated with GMD. We found 146 genes in Class 1 and 199 genes in Class 2, which have negative effect and positive effect for GMD, respectively. The crossover of genes correlated with functional changes and those correlated with structural changes in MDD is shown in Fig. 3A, and the full list is given in Supplementary Table 8. Two typical genes in Classes 1 and 2, coiled-coil domain-containing protein 39 (*CCDC39*) and chromosome X open reading frame 57 (*CXorf57*), are illustrated in Fig. 3B.

### Comparison of depression-related genes with differentially expressed genes in post-mortem brains and risk genes identified by GWAS

We compared depression-related genes with the transcriptional downregulated genes in post-mortem cortex of patients with MDD using analytic data from previous reports^28^ (see the Materials and methods section). Compared with control genes not correlated with the functional or structural changes in MDD, downregulated genes in the post-mortem brain samples of the hippocampus, pre-frontal cortex and associated striatum account for a larger percentage of the genes in Class 1 and a lower percentage of the genes in Class 2 (Fig. 3C). This result indicates that transcriptional correlates of *in vivo* depression cortical phenotypes can capture patterns of *ex vivo* gene downregulation in patients to some extent.

Furthermore, we compared depression-related genes with the risk genes associated with depression identified by GWAS^31^ and found seven depression-related genes have genetic associations with MDD, including five genes in Class 1 and two genes in Class 2 (see the Materials and methods section). The percentage of depression risk genes occupying the genes in Class 1 (2.51%) is higher than that occupying the genes in Class 2 (1.37%), and both of them are higher than that occupying control genes (1.32%), as shown in Fig. 3D. The top significant risk genes related with MDD are *DENND1* in Class 1 and *ZNF165* in Class 2, with their Howard *P*-values 1.54 × 10^−14^ and 1.95 × 10^−12^, respectively.

### Network topological property analysis of depression-related gene products

**Figure 4:**
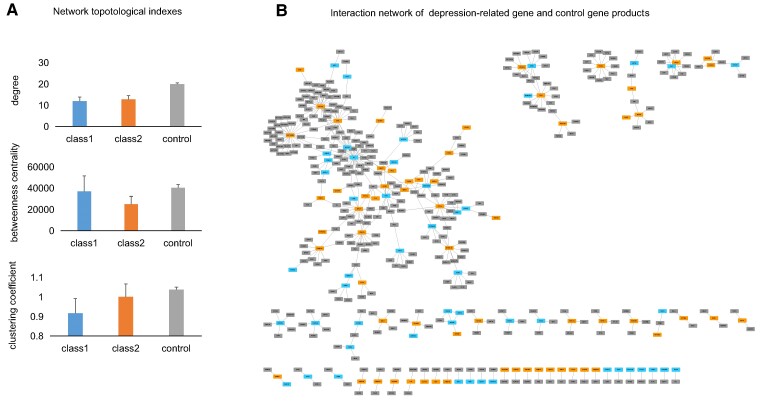
**The network properties and interaction network of the depression-related gene products.** (**A**) The network properties of depression-related gene and control gene products. The number of genes in Class 1, Class 2 and controls is 146, 199 and 4089, respectively. (**B**) The interaction network of the depression-related gene products. The size of a node is proportional to its network degree. This network is visualized by the tool Cytoscape.^39^

To investigate how depression-related genes interact with each other, we downloaded the protein interactions in the STRING database (https://string-db.org/, version 11.5) with high scores (combined_score > 900) and computed three typical topological indices of depression-related gene products in the interaction network, including degree, betweenness centrality and clustering coefficient (see the Materials and methods section and Supplementary Table 9). As shown in Fig. 4A, the degree, betweenness centrality and clustering coefficient of the genes in Class 1 are significantly lower than those in Class 2 and the control genes. This indicates that depression-related gene products tend to interact with few other proteins, have lesser influence over the spread of information through the network and involve few clusters, than control gene products. This result is in accord with a previous report that differentially expressed genes in MDD tend to locate on the periphery of co-expression networks.^38^ The interaction network of the depression-related gene products is demonstrated in Fig. 4B.

### Temporal–spatial expression specificity and functional annotation analysis of depression-related genes

**Figure 5:**
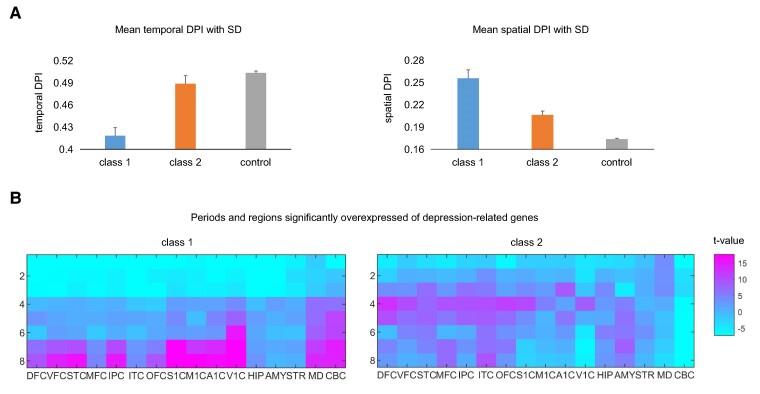
**The temporal–spatial expression specificity of depression-related genes.** (**A**) The mean temporal and spatial DPI values of depression-related genes and control genes. The number of genes in Class 1, Class 2 and controls is 146, 199 and 4089, respectively. (**B**) The periods and regions in which depression-related genes significantly overexpressed. The *t*-values for *t*-tests of the expression of depression-related genes compared with those of control genes were mapped to the periods and brain regions. The corresponding *t*-values and *P*-values for *t*-tests were available in Supplementary Table 11. A1C, primary auditory cortex; AMY, amygdala; CBC, cerebellar cortex; DFC, dorsolateral pre-frontal cortex; DPI, dispersion index; HIP, hippocampus; IPC, posteroventral parietal cortex; ITC, inferolateral temporal cortex; M1C, primary motor cortex; MD, mediodorsal nucleus of the thalamus; MFC, anterior cingulate cortex; OFC, orbital frontal cortex; S1C, primary somatosensory cortex; STC, posterior superior temporal cortex; STR, striatum; V1C, primary visual cortex; VFC, ventrolateral pre-frontal cortex.

To explore the temporal–spatial expression characteristics of depression-related genes, we retrieved the gene expression data from the developing human brains, available through the Allen Brain Atlas,^35^ comprising 547 transcriptome samples across 16 brain regions during 8 developmental periods. Utilizing the measure DPI,^36^ we quantitatively assessed the expression specificity of depression-related genes across different periods and regions (for details, refer the Materials and methods section). As depicted in Fig. 5A, depression-related genes exhibit higher spatial DPI values and lower temporal DPI values compared with the control genes (*P*-values for two-sided *t*-test <0.001). This indicates that MDD may induce functional and structural alterations in limited brain regions across multiple periods.

We identified the developmental periods and brain regions where depression-related genes were significantly highly expressed relative to the control genes, as determined by the two-sided *t*-test, illustrated in Fig. 5B. Genes categorized in Class 1 were significantly overexpressed in regions such as VFC, STC, IPC, OFC, S1C, M1C, A1C, V1C, MD and CBC compared with the control genes (*P* < 0.001). Notably, the genes in Class 1 were significantly highly expressed in CBC and V1C from childhood to adulthood, while highly expressed in other relevant brain regions primarily observed during adolescence and adulthood. These findings suggest that early MDD phenotypes might manifest in CBC and V1C. In contrast, the genes in Class 2 demonstrated significant overexpression in MFC, ITC, HIP and AMY, which has been extensively reported by prior studies.^5,40^ This suggests that MDD may lead to diminished functional activity levels in these brain regions.

**Figure 6:**
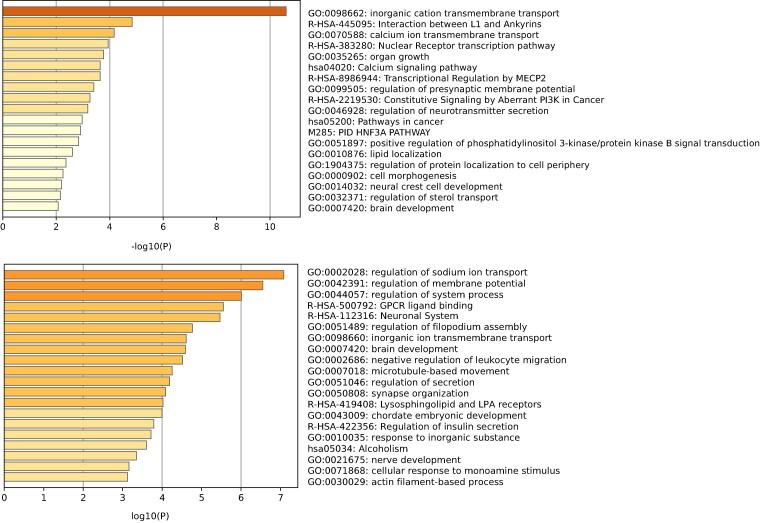
Enriched ontology clusters of depression-related genes.

Furthermore, we employed the gene annotation and analysis resource Metascape^41^ to analyse the function annotations and biological pathways of depression-related genes. We used gene ontology (GO) to enrich these genes for specific molecular functions, biological processes and cellular components, and consulted the Kyoto Encyclopaedia of Genes and Genomes to identify pertinent biological pathways. We calculated cumulative hypergeometric *P*-values following multi-test corrections and determined enrichment factors for filtering purposes. The most enriched functional annotation terms for the depression-related genes are displayed in Fig. 6, with the complete list provided in Supplementary Table 10. There are significant differences between the function annotations enriched by the genes in Class 1 and those in Class 2. The genes in Class 1 are enriched for the GO biological processes of inorganic cation transmembrane transport (*P* = 2.51 × 10^−11^) and calcium ion transmembrane transport (*P* = 6.76 × 10^−5^), while the genes in Class 2 are enriched for the GO biological processes of regulation of sodium ion transport (*P* = 8.32 × 10^−8^) and regulation of membrane potential (*P* = 2.82 × 10^−7^).

### Sex difference analysis in the expression of depression-related genes

**Figure 7:**
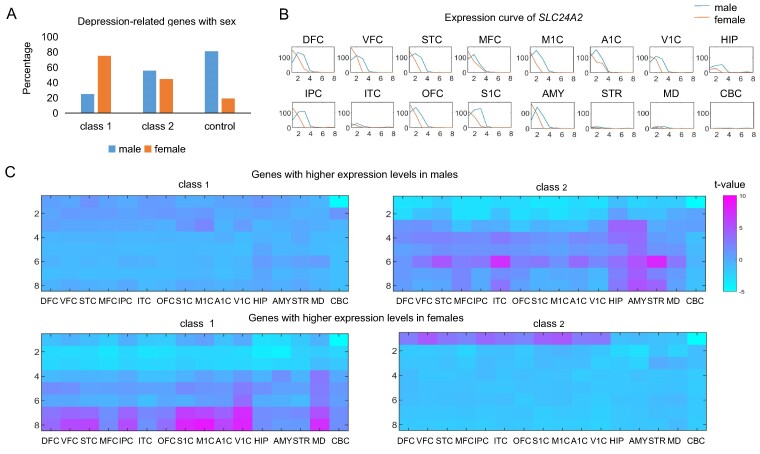
**Sex differences in the expression of depression-related genes.** (**A**) The percentage of the genes highly expressed in males or females occupying depression-related genes with sex difference. The number of genes with sex difference in Class 1, Class 2 and controls is 8, 9 and 279, respectively. (**B**) The expression change curve of *SLC24A2* varying with periods in different regions. (**C**) The periods and regions in which depression-related genes with sex difference are overexpressed. The corresponding *t*-values and *P*-values are available in Supplementary Table 11. The number of genes in Class 1, Class 2 and controls is 146, 199 and 4089, respectively. A1C, primary auditory cortex; AMY, amygdala; CBC, cerebellar cortex; DFC, dorsolateral pre-frontal cortex; HIP, hippocampus; IPC, posteroventral parietal cortex; ITC, inferolateral temporal cortex; M1C, primary motor cortex; MD, mediodorsal nucleus of the thalamus; MDD, major depressive disorder; MFC, anterior cingulate cortex; OFC, orbital frontal cortex; S1C, primary somatosensory cortex; STC, posterior superior temporal cortex; STR, striatum; V1C, primary visual cortex; VFC, ventrolateral pre-frontal cortex.

We examined the sex differences in the expression of depression-related genes using gene expression data from developing human brains. Through two-sided *t*-tests on the expression data of the males and females through all periods, we identified 17 depression-related genes with sex differences in expression (*P* < 0.01), including 8 genes in Class 1 and 9 genes in Class 2 (see the Materials and methods section). Then, we counted the percentage of genes highly expressed in males and females occupying the depression-related genes with sex differences, as shown in Fig. 7A. Genes highly expressed in females accounted for a higher proportion in the depression-related genes with sex differences than those highly expressed in males, compared with the control genes. Especially, the genes in Class 1 tend to be more highly expressed in females than males, which may be associated with the higher incidence rate of depression in female patients.^42,43^ The solute carrier Family 24 Member 2 (*SLC24A2*) in Class 1 was found to have the most expression differences between males and females (*P* = 1.19 × 10^−4^), as shown in Fig. 7B. According to functional annotations, *SLC24A2* is involved in calcium ion import, long-term synaptic depression and calcium ion import across plasma membrane.

Furthermore, we explored the periods and regions where depression-related genes with sex differences are significantly overexpressed, as depicted in Fig. 7C. The genes in Class 1 predominantly show increased expression in DFC, VFC, STC, IPC, OFC, S1C, M1C, V1C, A1C and MD in females compared with in males during adolescence and adulthood. The differential expression of depression-related genes between males and females may underlie the sex differences in MDD varied with age groups.^44,45^

## Discussion

In this study, we uncovered several crucial aspects of gene signatures linked to brain structural and functional alterations in MDD, including their network properties, temporal–spatial expression specificity, molecular function and biological process and sex differences. First, we simultaneously detected inter-group differences between patients with MDD and healthy controls using fMRI and MRI data, and identified gene signatures correlated with functional or structural changes in MDD through the association analysis of gene expression and brain imaging. We observed that depression-related genes tend to exhibit differential expression in post-mortem cortex samples of patients with MDD and are enriched in risk genes identified by GWAS. Secondly, we evaluated the network properties, functional annotations and the temporal–spatial specificity of depression-related genes. Compared with control genes, depression-related genes tend to be located on the periphery of the interaction network, are enriched in the process of ion transmembrane transport and display higher spatial specificity and lower temporal specificity in expression. Lastly, we investigated the sex differences of depression-related genes expressed in different periods and regions, noting a trend of overexpression of depression-related genes in multiple brain regions in females compared with in males. These findings can advance our understanding of the pathogenesis and sex differences of MDD from the perspective of gene expression.

The progression of MDD is accompanied by a series of structural and functional alterations in the brain. Previous analyses typically focused on one aspect of the symptoms caused by MDD, such as structural changes or functional changes.^18,19,46-48^ In this study, we simultaneously examined the structural and functional changes between patients with MDD and healthy controls based on fMRI and MRI. We discovered that MDD can induce extensive functional and structural changes, and there is a close correlation between structural and functional changes in MDD. The decrease of GMD in specific brain regions is partially accompanied by the enhancement of functional activity, while the increase of GMD is partially accompanied by the weakening of functional activity. This result is consistent with the previous multimodal meta-analysis of MDD,^5^ which revealed increased grey matter volume with decreased brain activity in the left lateral OFC, and decreased grey matter volume with increased brain activity in the right SMA.

Unlike the PLS analyses employed in previous research,^17,19^ which identified a linear combination of genes exhibiting a similar cortical pattern of expression to the map of volumetric changes observed in MDD, we determined gene signatures of MDD by associating the functional and structural changes in MDD with the gene expression profiles from human brain samples. We separated them by directional effects into genes with negative effect (Class 1) and positive effect (Class 2) for GMD. Comprehensive analysis shows that there are significant differences in network properties, temporal–spatial expression specificity and function annotations between genes in Classes 1 and 2. The genes in Class 1 have lower network degree, more specialized functions, higher spatial specificity, lower temporal specificity and more sex differences than genes in Class 2. Meanwhile, the genes in Class 1 account for a larger percentage of downregulated genes in the post-mortem brain samples and risk genes identified by GWAS than genes in Class 2. These findings remind us that it is necessary to carefully distinguish between genes with negative effect and positive effect for GMD, which may play different roles in the occurrence and development of depression. The differences between multiple attributes of genes with negative effect and control genes are much greater than those of genes with positive effect and control genes. Combining their different positioning in the interaction network, we speculated that the genes in Class 1 may be directly related with MDD, and the genes in Class 2 may be indirectly related with MDD, which adjust their expression levels to compensate for pathologically induced expression changes of the genes in Class 1. This result requires further experimental verification.

Comprehending the temporal–spatial specificity of gene expression in the human brain is instrumental in elucidating the neurodevelopment, sexual dimorphism and heightened vulnerabilities to specific brain disorders.^22,49^ Utilizing the developmental data from human brains, we examined the periods and regions where depression-related genes are significantly overexpressed by introducing the specificity measure of gene expression. We observed significant overexpression of depression-related genes in regions such as VFC, STC, IPC, OFC, S1C, M1C, A1C, V1C, MD and CBC compared with control genes during both adolescence and adulthood. Notably, the majority of depression-related genes exhibit elevated expression levels in adulthood relative to adolescence, which could account for differing manifestations of MDD symptoms between these life stages.^50,51^ Concurrently, we identified significant disparities in the expression patterns of depression-related genes between males and females. The genes in Class 1 manifest overexpression across multiple regions, including VFC, STC, IPC, OFC, S1C, M1C, A1C and MD, in females than in males. These findings could enrich our understanding of the sex differences in the onset and progression of MDD and may inform the development of tailored diagnostic and therapeutic interventions for patients with MDD of different genders.

There were several limitations to this work. First, the reliance of gene expression profiles from healthy human brains in AHBA to interpret structural and functional changes is constrained by the potential variation in transcriptional activity between patients and healthy individuals. Secondly, due to the sampling precision of the BrainSpan Atlas, our analysis was confined to the temporal–spatial expression attributes of depression-related genes within 8 periods and 16 brain regions. The validity of our findings needs to be confirmed through alternative transcriptome atlases as higher resolution expression data sets become accessible. Finally, it is imperative to underscore that the collaborative mechanisms through which these genetic signatures contribute to the pathogenesis of MDD warrant further investigation, given that MDD is a complex disorder influenced by multiple genes.

## Conclusion

In this paper, we identified gene signatures with different directional effects for functional and structural alternations in MDD and investigated their temporal–spatial expression specificity, network properties, function annotation and sex differences systematically. We found 916 gene signatures significantly correlated with functional and structural brain image alterations in MDD. These signatures are enriched in differentially expressed genes in post-mortem cortex samples of patients with MDD and risk genes identified by GWAS. We found the genes with a negative effect on GMD possess lower network degree, more specialized functions, higher spatial specificity, lower temporal specificity and more sex differences compared with genes with a positive effect. The differences between various attributes of genes with negative effect and control genes are much greater than those of genes with positive effect and controls genes. Drawing upon the gene expression data from developing human brains available in AHBA, we observed that depression-related genes exhibit higher spatial specificity and lower temporal specificity than control genes. This suggests that MDD may emerge at multiple periods, but only induces functional and structural changes in specific brain regions. Concurrently, we noted a tendency for depression-related genes to be more highly expressed in females than in males. These findings enhance our comprehension of the pivotal roles gene expression plays in the emergence and progression of MDD, as well as the unique expression patterns of MDD gene signatures in multiple attribute dimensions.

## Supplementary Material

fcae258_Supplementary_Data

## Data Availability

The neuroimaging data used in this research were obtained from the REST-meta-MDD project (http://rfmri.org/REST-meta-MDD). The expression data employed in this study are publicly accessible through the Allen Institute for Brain Atlas (see http://brain-map.org/). Gene expression profiles of developing human brains are sourced from the Allen Brain Atlas (http://www.brainspan.org). The cortical expression data of post-mortem brain tissues, including pre-frontal cortex, hippocampus and striatum, from control subjects and well-matched subjects with MDD are deposited in GEO (GSE53987). The inventory of risk genes associated with depression identified by GWAS can be found in the supplementary table of the article with PMC6522363. Information on protein interactions in humans was derived from the STRING database (https://string-db.org/, version 11.5) with high scores (combined_score > 900). The authors affirm that the data underpinning the results presented in this study are included within the manuscript and its supplementary files and are available upon request.
