# Supplementary material for: Gene expression and brain imaging association study reveals gene signatures in major depressive disorder
Source: Brain Commun. 2024 Aug 13;6(4):fcae258. doi: 10.1093/braincomms/fcae258 (PMC11342243; doi:10.1093/braincomms/fcae258)
Supplement: fcae258_Supplementary_Data [file fcae258_supplementary_data.zip › Supplementary_Table_3.pdf]

Supplementary Table 3: Periods of human development and adulthood as defined in this study.

Period	Description	Age	Sample number
**1**	Early fetal	Age<14pcw	8 (4 males, 4 females)
**2**	Middle fetal	14pcw≤Age<24pcw	7 (4 males, 3 females)
**3**	Late fetal	24pcw≤Age< Birth	3 (1 males, 2 females)
**4**	Infancy	Birth≤Age<1yrs	4 (3 males, 1 females)
**5**	Early childhood	1yrs≤Age<6yrs	5 (2 males, 3 females)
**6**	Middle and late childhood	6yrs≤Age<12yrs	3 (2 males, 1 females)
**7**	Adolescence	12yrs≤Age<20yrs	4 (2 males, 2 females)
**8**	Adulthood	20yrs≤Age	7 (4 males, 3 females)
