# Supplementary material for: Gene expression and brain imaging association study reveals gene signatures in major depressive disorder
Source: Brain Commun. 2024 Aug 13;6(4):fcae258. doi: 10.1093/braincomms/fcae258 (PMC11342243; doi:10.1093/braincomms/fcae258)
Supplement: fcae258_Supplementary_Data [file fcae258_supplementary_data.zip › Supplementary_Table_4.pdf]

Supplementary Table 4 Ontology and nomenclature of analyzed brain regions.

Category	Structure id	Structure name
Neocortex	M1C	primary motor cortex (area M1, area 4)
	S1C	primary somatosensory cortex (area S1, areas 3,1,2)
	A1C	primary auditory cortex (core)
	V1C	primary visual cortex (striate cortex, area V1/17)
	DFC	dorsolateral prefrontal cortex
	VFC	ventrolateral prefrontal cortex
	OFC	orbital frontal cortex
	IPC	posteroventral (inferior) parietal cortex
	STC	posterior (caudal) superior temporal cortex (area 22c)
	ITC	inferolateral temporal cortex (area TEv, area 20)
	MFC	anterior (rostral) cingulate (medial prefrontal) cortex
Archicortex	HIP	hippocampus
	AMY	amygdala
	STR	striatum
	MD	mediodorsal nucleus of the thalamus
Subcortex	CBC	cerebellar cortex
