# Supplementary material for: Gene expression and brain imaging association study reveals gene signatures in major depressive disorder
Source: Brain Commun. 2024 Aug 13;6(4):fcae258. doi: 10.1093/braincomms/fcae258 (PMC11342243; doi:10.1093/braincomms/fcae258)
Supplement: fcae258_Supplementary_Data [file fcae258_supplementary_data.zip › Supplementary_Table_7.pdf]

Supplementary Table 7 (part 1) Regions with significant group differences in ALFF

Index	Name	BA	Cluster size	Coordinates of peak voxel	T value of peak voxel
1	Supp_Motor_Area_R	6	23871	3, 15, 63	-5.2145
2	Cerebellum_9_L	\	10	-13, -53, -43	-2.9803
3	Temporal_Pole_Sup_L	\	7	-45, 9, -24	-3.1237
4	Temporal_Mid_L	22	9	-63, -54, 9	-3.1846
5	Temporal_Sup_L	22	5	-60, -42, 21	-3.454

Supplementary Table 7 (part 2) Regions with significant group differences in fALFF

Index	Name	BA	Cluster size	Coordinates of peak voxel	T value of peak voxel
1	Cingulate_Post_L	30	5	0, -54, 21	-2.8193
2	Precuneus_L	31	5	-3, -60, 30	-2.4582
3	Paracentral_Lobule_R	5	32	3, -39, 66	-3.497

Supplementary Table 7 (part 3) Regions with significant group differences in ReHo (Cluster Size>30)

Index	Name	BA	Cluster size	Coordinates of peak voxel	T value of peak voxel
1	Temporal_Pole_Mid_R	38	33	45, 9, -27	-4.3249
2	Cerebellum_4_5_L	\	95	-3, -60, -12	-4.063
3	Fusiform_L	37	152	-39, -63, -18	-4.9898
4	Occipital_Mid_L	19	84	-48, -72, 3	-4.9914
5	Occipital_Sup_R	\	289	24, -60, 36	-4.7469
6	Thalamus_L	\	30	-9, -18, 9	-4.3357
7	Cingulate_Post_L	31	105	-3, -48, 30	-4.5275
8	Angular_L	39	40	-42, -63, 33	-4.159
9	Occipital_Mid_L	\	196	-27, -78, 24	-4.673
10	Cingulate_Mid_R	23	45	6, -21, 30	-4.0706
11	SupraMarginal_R	2	38	51, -30, 39	-4.4309
12	Postcentral_R	3	196	36, -33, 60	-4.5698

Supplementary Table 7 (part 4) Regions with significant group differences in GMD (Cluster Size>200)

Index	Name	BA	Cluster size	Coordinates of peak voxel	T value of peak voxel
1	Temporal_Pole_Sup_L	38	751	-31.5, 18, -30	-6.1731
2	Temporal_Mid_L	\	202	-43.5, 4.5, -30	-7.2373
3	Temporal_Inf_R	\	287	40.5, -54, -7.5	-5.7257
4	Temporal_Sup_R	\	425	45, -24, -1.5	-7.2093
5	Frontal_Inf_Oper_R	45	228	55.5, 16.5, 6	-5.5112
6	OFCmed_L	\	428	-15, 42, -18	-6.9558

7	Insula_L	\	461	-25.5, 25.5, 7.5	-8.2081
8	Caudate_R	\	1254	7.5, 13.5, 9	-7.7321
9	Calcarine_L	30	208	-22.5, -63, 9	-7.2134
10	SupraMarginal_R	40	497	57, -36, 30	-7.3503
11	Frontal_Sup_Medial_L	8	202	-1.5, 39, 49.5	-5.6468
12	Postcentral_R	3	424	37.5, 24, 48	-7.7211
13	Frontal_Sup_2_R	6	394	25.5, -4.5, 70.5	-5.6308
