# Supplementary material for: Gene expression and brain imaging association study reveals gene signatures in major depressive disorder
Source: Brain Commun. 2024 Aug 13;6(4):fcae258. doi: 10.1093/braincomms/fcae258 (PMC11342243; doi:10.1093/braincomms/fcae258)
Supplement: fcae258_Supplementary_Data [file fcae258_supplementary_data.zip › supplementary_table_legends.docx]

**Supplementary Tables - Legends**

**Supplementary Table 1:** The list of differentially expressed genes in postmortem brain samples

**Supplementary Table 2:** Depression-related genes found in risk depression genes identified by GWAS

**Supplementary Table 3**: Periods of human development and adulthood as defined in this study

**Supplementary Table 4**: Ontology and nomenclature of analyzed brain regions

**Supplementary Table 5**: The temporal and spatial DPI values of the depression-related genes

**Supplementary Table 6**: Depression-related genes with sex differences

**Supplementary Table 7:** The regions with significant group differences in each of the functional indices and GMD

**Supplementary Table 8**: Depression-related gene list

**Supplementary Table 9:** Network topological properties of depression-related gene products

**Supplementary Table 10**: Functional annotation terms of depression-related genes

**Supplementary Table 11**: The t-values and p-values of periods and regions in which depression-related genes are overexpressed
